# The Impact of Equine-Assisted Therapy on Equine Behavioral and Physiological Responses

**DOI:** 10.3390/ani9070409

**Published:** 2019-07-01

**Authors:** Tiago Mendonça, Cécile Bienboire-Frosini, Fanny Menuge, Julien Leclercq, Céline Lafont-Lecuelle, Sana Arroub, Patrick Pageat

**Affiliations:** 1 Behavioral and Physiological Mechanisms of Adaptation Department, Research Institute in Semiochemistry and Applied Ethology, IRSEA, 84400 Apt, France; 2 Animal Experimentation Department, IRSEA, 84400 Apt, France; 3 Statistical Analysis Department, IRSEA, 84400 Apt, France; 4 Semiochemicals’ Identification and Analogs’ Design Department, IRSEA, 84400 Apt, France

**Keywords:** behavior, emotions, horse, equine-assisted therapy, heart rate variability, welfare

## Abstract

**Simple Summary:**

Equine-assisted therapies (EATs) are often applied to patients with different types of either mental or physical conditions. The increasing application of EATs has induced interest in the scientific community about equine well-being during these therapies. This study aimed to investigate whether equine-assisted therapy (EAT) creates negative or positive emotions in horses and the influence of patients’ therapy expectations (one group of patients had physical and psychological expectations, and one group of patients had only psychological expectations) on horses’ behavioral and physiological responses. We observed 58 pairs (patient–horse) during EAT sessions and when the horses were at rest. Then, we compared the horses’ behavioral and physiological responses between the different periods of therapy and among the groups of patients. Our results suggested that the EAT in this study was neither a negative nor a positive event. EATs with patients who had both physical and psychological expectations were more challenging for horses than those with patients who had only psychological expectations. Further research on EAT should focus on providing horses with positive stimulation and reinforcement to understand whether a positive association with EAT can be created.

**Abstract:**

Equine-assisted therapies (EATs) have been widely used in the treatment of patients with mental or physical conditions. However, studies on the influence of equine-assisted therapy (EAT) on equine welfare are very recent, and the need for further research is often highlighted. The aim of this study was to investigate whether EAT creates negative or positive emotions in horses, and the influence of patients’ expectations (one group of patients had physical and psychological expectations and one group of patients had only psychological expectations) on horses’ emotional responses. Fifty-eight pairs (patient–horse) were involved in this study. Behaviors and heart rate variability (HRV) data were collected during a resting phase, a preparation phase in which the patients brushed and saddled the horse, and a working phase. Behaviors and HRV were compared between phases and among the groups of patients. Our results suggested that the EAT in this study was neither a negative nor a positive event. EATs with patients who had both physical and psychological expectations were more challenging for horses than those with patients who had only psychological expectations. Further research should focus on providing horses with positive stimulation and reinforcement to understand whether a positive association with EAT can be achieved.

## 1. Introduction

Animal-assisted interventions (AAIs) is a term that is commonly used to describe any modality using animals to improve the physical, mental, and social conditions of humans [1]. These interventions have proven effects in applications for autism spectrum disorder, Alzheimer’s disease, behavioral problems, and emotional well-being [1,2,3]. Other medical issues in which this therapy has been applied include Parkinson’s disease [4], heart failure [5], cerebral palsy [6], and neurological impairments and applications for children with developmental delays [7].

AAIs that involve equines are usually defined as equine-assisted therapies (EATs) or equine-assisted activities (EAAs) [8]. EATs have been used to improve social, emotional, and physical domains in patients suffering from anxiety, depression, autism spectrum disorder, multiple sclerosis, and spinal cord injury or to improve balance, muscle symmetry, coordination, and posture [6,8,9].

Animal welfare has become a major concern in all activities that involve animals, including animal production [10], sports [11], work [12] and even pet ownership [13]. The awareness of the welfare of the animals used in EATs is a focus of interest to the scientific community along with its applications [14]. The most commonly used physiological indicators of animal welfare focus on the negative impact of an activity on animal welfare, assuming the absence of a negative impact to be associated with well-being or “positive welfare” [15]. However, the definition of positive welfare includes not only the absence of negative experiences or feelings, but also the presence of positive experiences or feelings [16]. This means that the study of animal welfare should include both negative and positive indicators.

Equine-assisted therapy (EAT) activities that involve horseback riding appear to be no more stressful for horses than recreational horseback riding activities [14,17]. Additionally, in a previous study, interactions between patients experiencing post-traumatic stress disorder and horses assisting in their therapy were found to be equally stressful as the interactions between healthy humans and horses [18]. The authors of this study suggested that horses respond more to physical cues than to emotional cues from humans [18].

Many studies of EATs that have investigated behavioral and/or physiological aspects have suggested that EAT activities do not produce negative outcomes, such as stress, in horses [18,19]. Considering the absence of negative outcomes, some authors have suggested that EAT may produce positive outcomes for horses [14]. However, one study that investigated both negative (plasmatic cortisol concentrations and heart rate variability) and positive (plasmatic oxytocin concentrations) outcomes of EAT found no difference between the parameters [20]. Nevertheless, there are relatively few studies on EATs, and only one investigated positive outcomes of these activities.

Some authors have demonstrated that heart rate variability (HRV) is a suitable tool to study equine emotional responses in animals involved in EAT [21]. HRV analysis the autonomic nervous system (ANS) activity [22]. The frequency domain analysis of HRV studies the sympathetic (SNS) and parasympathetic (PNS) components of the ANS [23]. This analysis includes parameters, such as low frequency (LF), high frequency (HF), and very low frequency (VLF); these are influenced by the respiratory rate, which varies according to species; therefore, the frequency band widths should be adapted in this type of analysis [24,25].

Each of the described HRV parameters reflects different aspects of ANS activity [25]. Some authors have suggested that LF may represent sympathetic activity [22,26]. HF is commonly considered a marker of the cardiac vagal tone, and consequently, the parasympathetic system [22,23,27]. The LF/HF ratio reflects the SNS–PNS balance [22,23,27]. The VLF component of HRV has been associated with a slow recovery of the ANS balance after a mental or physical effort [28]. The analysis of the ANS activity provides an objective tool to study both positive and negative outcomes of EATs [16,29].

The emotional states are best assessed by a combination of behavioral and physiological measures [30]. Physiological and behavioral indicators should include both positive and negative outcomes for the assessment of equine emotional states and welfare [16]. Some behaviors have been associated with negative emotions [14,19], while others have been associated with positive emotions [31]. Thus, these behaviors may be useful as negative and positive indicators of equine emotions in association with physiological indicators.

The aim of this study was to investigate whether EAT creates negative or positive emotions in horses and the influence of patients’ therapy expectations on horses’ emotional responses.

## 2. Materials and Methods

This project was approved by the Research Institute in Semiochemistry and Applied Ethology (IRSEA) Ethics Committee (C2EA125) and the French Ministry of Research (APAFIS Process number 11949).

General and specific information about the patients and the horses was collected through a questionnaire survey. Patients’ medical conditions and expectations were collected with the agreement of the patients and/or their families. Nevertheless, to respect patient confidentiality, the diagnoses and expectations of individual patients were not reported; instead, data were classified into different categories.

This study was conducted in Avignon, France, between March and July 2018.

### 2.1. Population

This study included nine horses (10 ± 3 years old) and 51 patients (18 ± 11 years old) who participated in EATs.

The animals were recruited from two different equestrian facilities. Housing conditions were the same in both centers. Horses lived in paddocks that they shared with other conspecifics. The population included four females and five geldings (six grade horses, one Mérens, one Halfinger, and one Carmargue). These horses have been involved in EAT for: 9 years; 8 years; 6 years; 4 years; two horses for 3 years; 2 years; 1 year; and 6 months.

The patients involved in this study have been diagnosed with different medical conditions (see Table 1). From the patients’ and therapists’ perspectives, the expectations for improvement related to these therapies could be divided into two categories: psychological (PSY) or both physical and psychological (PHY + PSY) expectations.

The statistical unit considered for this study was the pair (patient + horse). Some of the included patients had therapy with different horses on different days. Fifty-eight pairs of patient–horse were involved in this study.

### 2.2. Study Design and Data Collection

This investigation included a resting phase and two phases of EATs (preparation and working phases).

The resting phase consisted of the collection of HRV data in the normal living and husbandry conditions of the horses.

The preparation phase consisted of controlled interactions between the patient and the horse, such as petting, brushing, saddling, cleaning the horses’ feet, and feeding or watering the horses. These interactions differed according to the patients’ needs and abilities, and were selected by the therapists. Preparative sessions were performed near the paddocks.

The working phase followed the preparation phase. The patients could either ride or perform in-hand training with the horses. In-hand training included the following: the approach and the initial contact with the horse, making the horse move forward or backward, leading the horse to cross obstacles or pass parallel bars on the ground, and lunging the horse in a round pen. The therapists made the decision of whether the patient should ride or perform in-hand training. Working sessions were performed in riding arenas of variable size.

Each phase lasted between 20–30 minutes, and took place between 09:00–13:00 and 14:00–16:00 during the day.

Behavior data were collected for both the preparation and working phases by one observer through direct observation. The considered behaviors included “ears pinned back” (EPB), “head lateral movement” (HLM), “snort”, and “defecation” (see Table 2) [14,31]. These parameters were assessed in terms of frequency.

HRV data were collected during all the phases with Polar V800 Equine^®^ equipment (Kemple, Finland). Then, the collected data were transformed with Kubios^®^ software (Kuopio, Finland), and only normal R-R intervals were considered for the HRV analysis. The described data included time domain (heart rate) and frequency domain analysis (see Table 3). Frequency band thresholds were established within each frequency interval using the following parameters: LF power = 0.005–0.07 Hz; HF power = 0.07–0.5 Hz; VLF power = 0.001–0.005 [25].

### 2.3. Statistical Analysis

Statistical analysis was carried out using SAS 9.4 software Copyright © 2002-2012 by (SAS Institute Inc., Cary, NC, USA). The significance threshold was classically fixed at 5%.

#### 2.3.1. Data

The duration of each phase differed according to the patient’s responses to the therapy. For horse behaviors, the proportion of the frequency over the duration of the given phase was calculated to standardize this variable among different phases. Finally, the frequency of each behavior per minute (freq/min) was considered for the statistical analysis.

HRV data were analyzed as raw data to compare the differences between the two EAT phases and between the two categories of patients’ therapy expectations. Then, the differences (Δ) between the preparation and resting phases and between the working and resting phases were calculated to study the evolution of these parameters from resting to the two different EAT situations. These parameters were defined as ΔHR, ΔVLF, and ΔLF/HF ratio.

#### 2.3.2. Statistical procedures

For HR and the LF/HF ratio, the assumption of normality of the data was tested using residual diagnostics plots and the UNIVARIATE procedure. Comparisons between phases and between patients’ expectations were performed with the general linear mixed model using the MIXED procedure (two-factor analysis). When significant differences were found, multiple comparisons were analyzed with Tukey’s test using the LSMEANS statement.

For EPB, HLM, and VLF, the assumption of normality of the data was tested with the above-described procedure, although the assumption was not verified. Then, box-cox transformation was performed. The assumption of normality of the data was retested using residual diagnostics plots and the UNIVARIATE procedure. Comparisons between phases and between patients’ expectations were performed with the general linear mixed model using the MIXED procedure. When significant differences were found, multiple comparisons were analyzed with Tukey’s test using the LSMEANS statement.

For the differences in the cardiac parameters (ΔHR; ΔVLF; ΔLF/HF ratio), the assumption of normality of the data was tested with the above-described procedure, although the assumption was not verified. Then, the data were analyzed with the Wilcoxon Signed-rank’s test (for paired samples).

For snort and defecation frequencies, data were transformed into binary variables, because the animals did not perform this behavior very often. In this case, the following code was adopted: 1 – the behavior was observed; 0 – the behavior was not observed. Comparisons between phases and patients’ expectations were performed with the generalized linear mixed model using the GLIMMIX procedure. When significant differences were found, multiple comparisons were analyzed with Tukey’s test using the LSMEANS statement.

## 3. Results

A few patients could not participate in one of the phases, as participation was not recommended due to their medical state, which lead to an absence of data. The lack of data concerned three patients in the preparation phase and one patient in the working phase.

One patient was highly disturbed by the presence of the observers during the preparation phase, so the therapists decided to remove the observers from the patient’s visual field. Therefore, it was not possible to observe the horse’s behavior during this phase.

One session occurred during feeding time. This caused a distraction for the horse, which was looking at the animal keepers feeding the animals, significantly increasing the HLM parameter. Therefore, the data concerning this parameter in the preparation phase were removed from the analysis.

In one working phase, a side walker was required to ensure the patient’s safety. This unexpected situation clearly modified the horse’s behavior. As some behavioral or HRV parameters were influenced by this condition, the authors decided to remove them from the statistical analysis (EPB, HLM, LF/HF ratio, and ΔLF/HF ratio).

Due to technical issues related to the heart rate monitor, the HRV data of two horses in the working phase were not recorded.

### 3.1. Behavior

There was no significant difference in EPB between the different phases, the two groups of patients’ therapy expectations, or the interaction of phase*patients’ therapy expectations (phases: DF = 1; F = 0.40; *p* = 0.53; patients’ therapy expectations: DF = 1; F = 0.13; *p* = 0.72; interaction of phase*patients’ therapy expectations: DF = 1; F = 1.64; *p* = 0.21).

**Figure 1:**
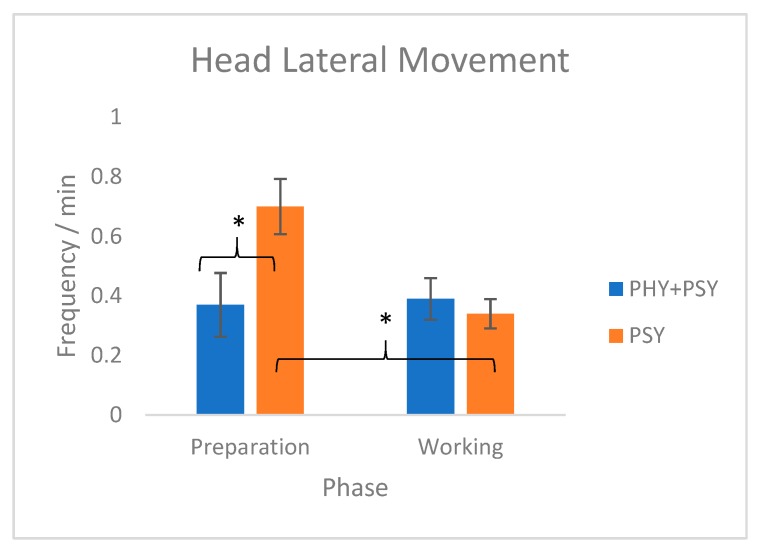
Equine-assisted therapies effect on Head Lateral Movement (freq./min.): effect of phase*patients’ therapy expectations. Mean ± standard error. * *p* < 0.05.

Significant differences were observed for HLM in the interactions between phases and patients’ therapy expectations (interaction of phase*patients’ therapy expectations: DF = 1; F = 6.60; *p* = 0.01). More precisely, in the preparation phase, horses performed on average two times more HLMs with the PSY group than with the PHY+PSY group. Additionally, horses involved in therapies with the PSY group performed on average two times more HLMs in the preparation phase than in the working phase (see Figure 1).

There was no significant difference in snort concerning either the different phases or the patients’ therapy expectations (phases: DF = 1; F = 0.05; *p* = 0.82; patients’ therapy expectations: DF = 1; F = 0.05; *p* = 0.82; interaction of phase*patients’ therapy expectations: DF = 1; F = 0.00; *p* = 0.99).

Defecation was only observed 11 times over a total of 111 EAT observations (preparation + working). For that reason, statistical analysis with a model was not applicable for this behavior.

### 3.2. HRV

HRV results concerning the interaction of phase*patients’ therapy expectations were not significant for any of the studied parameters (HR: DF = 1; F = 1.46; *p* = 0.23; VLF: DF = 1; F = 1.27; *p* = 0.27; LF/HF ratio: DF = 1; F = 0.56; *p* = 0.46).

#### 3.2.1. Phase Effect

Significant differences in HR, VLF, and the LF/HF ratio were observed between phases (HR: DF = 1; F = 76.67; *p* < 0.001; VLF: DF = 1; F = 4.59; *p* = 0.04; LF/HF ratio: DF = 1; F = 9.80; *p* < 0.01). More precisely, an increase of 10 beats per minute (bpm) on average in the horses’ HR between the preparation and working phases was observed. VLF and the LF/HF ratio were significantly higher in the working phase than in the preparation phase (see Table 4).

Significant differences in the ΔHR, ΔVLF, and ΔLF/HF ratio values were observed between phases (ΔHR: preparation: median = −3.000 VS working: median = 9.000; S = 570; *p* < 0.001; VLF: preparation: median = −0.020 VS working: median = 0.230; S = 264; *p* = 0.01; LF/HF ratio: preparation: median = −0.577 VS working: median = 1.516; S = 400; *p* < 0.001). More precisely, HR, VLF and the LF/HF ratio inversely changed from the resting phase to the other phases (see Table 4).

#### 3.2.2. Effect of Patients’ Therapy Expectations

**Figure 2:**
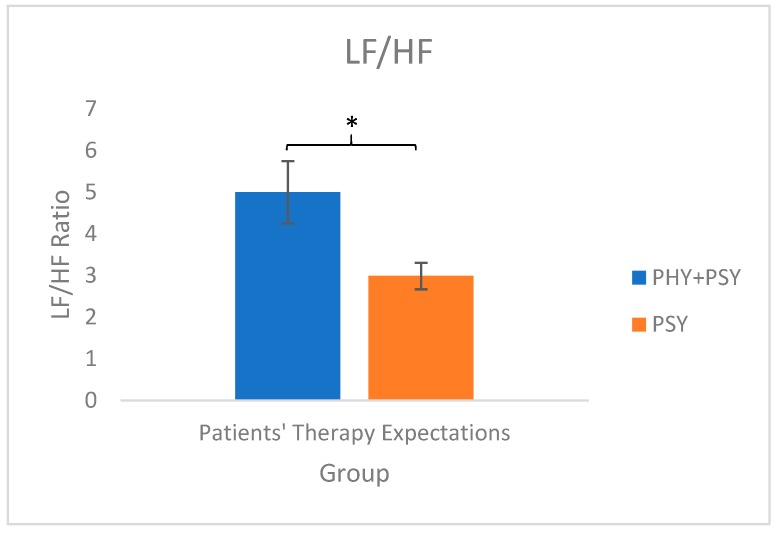
Effect of EAT on the LF/HF ratio: effect of patients’ therapy expectations. Mean ± standard error. * *p* < 0.05.

Significant differences were observed in the LF/HF ratio between the two groups of patients’ therapy expectations (patients’ therapy expectations: DF = 1; F = 5.83; *p* = 0.02). More precisely, the LF/HF ratio was higher in the PHY+PSY group than in the PSY group (see Figure 2). There were no significant differences in HR and VLF between the different groups of patients’ therapy expectations (HR: DF = 1; F = 1.74; *p* = 0.19; VLF: DF = 1; F = 0.42; *p* = 0.52).

## 4. Discussion

The results obtained with the different parameters showed that EAT may induce some behavioral and physiological changes in horses. Nevertheless, the results obtained regarding HRV, in particular LF/HF, were within the normal range in both phases [21]. Thus, these responses could not be linked to any stress-related response in the present study. Therefore, our results are in line with previous studies that have suggested that EAT does not increase stress [14,19,20].

### 4.1. Behavior

Considering the studied behaviors, the HLM frequency was lower in the PHY+PSY group than in the PSY group during preparation, suggesting that horses searched more for visual information [14] with the PSY group than with the PHY+PSY group. This result could be explained by the curiosity of horses about unusual types of interactions. In addition, a decrease in the HLM frequency from the preparation to the working phase was observed in the PSY group, suggesting that interactions required more visual contact for the horses during the preparation phase than during the working phase. Nevertheless, the observed frequencies of all the behaviors in this study were very low. In general, in this study, horses performed EPB and HLM less than one time per minute. Snort was only performed in approximately half of the observations. Defecation was observed in 11 out of 111 observations.

EPB and defecation, as well as HLM during riding sessions, are behaviors that are commonly associated with negative emotions [14,19]. Snort was previously considered a behavior related to arousal [32,33,34], but recently, this behavior has been associated with positive situations [31]. The relationship between each of these behaviors and either a positive or negative situation or emotions is interesting, because such relationships may indicate that the EAT activities in the present study were neither positive nor negative for the horses, as none of the behaviors were frequently expressed by the animals.

### 4.2. HRV

In terms of the HR, an increase of approximately 10 bpm between the preparation and the working phases was observed. VLF was less than 1% in the preparation phase, and a small increase from the preparation to the working phase was observed, although VLF was still less than 1% after the increase. Concerning the LF/HF ratio, an increase of approximately 1.8 times between the preparation phase and the working phase was observed. Additionally, the LF/HF ratio of the horses involved in EAT with the PHY+PSY group was approximately 1.7 times higher than that of the horses involved in EAT with the PSY group.

Regarding the Δ values, a decrease in HR of 3 bpm from the resting phase to the preparation phase and an increase of 9 bpm from the resting phase to the working phase were observed. Moreover, the VLF showed a small decrease (0.02) from the resting phase to the preparation phase, and a higher increase (0.23) from the resting phase to the working phase. Finally, a decrease in the LF/HF ratio from the resting phase to the preparation phase and an increase in the LF/HF ratio from the resting phase to the working phase were observed. Nevertheless, values of LF/HF appear to indicate physiological changes, and usual LF/HF values in EAT horses could be considered to range between 2–5 [21]. Besides the observed variability, the results of the present study concerning the LF/HF ratio were all within the above-described range.

Horses involved in other equestrian activities may show an increase in HR that can exceed 100 bpm during working/training sessions [35,36]. The EAT activities in this study were performed at a low intensity level, which explains the low levels of the HR changes observed in this study. Declining trends in the VLF component of HRV have been related to slow recovery of the ANS balance, and have also been associated with heart disorders [28]. However, in our study, we observed a short decrease in VLF in the preparation phase, which was immediately balanced during the working phase. An increase in the LF/HF ratio represents an increase in sympathetic activity, while a decrease in the LF/HF ratio represents an increase in parasympathetic activity [26,28]. In our study, an increase in this ratio was observed in the working phase, which could be associated with attempts by the horses to cope with the situation. In addition, patients in the PHY+PSY group induced higher LF/HF ratios than the patients in the PSY group. This suggests that therapies with patients in the PSY group were less challenging to the horses’ emotional state than therapies with patients in the PHY+PSY group. However, neither of the groups represented a major stressor to the horses. 

### 4.3. General Discussion

The benefits of EAT for people with mental and physical disabilities have been the subject of many investigations, which have demonstrated improvements in the muscle activity, social skills, and reductions in the anxiety of patients suffering from different medical conditions [6,8]. As EATs are getting popular, the welfare of horses involved in EATs has become a subject of increasing interest in the scientific community [14,19,20,21,37].

EAT activities are usually performed at low levels of physical activity [21], which was also observed in our study. Some authors have described HRV as a useful tool to study the emotional balance of horses involved in EAT and assess the horse–human partnership [21]. Indeed, our results support the use of HRV as an interesting tool to study equine perceptions of EAT because this analysis allows the study of the animal’s response toward a stimulus and its capacity to subsequently recover.

Investigations about EAT have suggested that these activities are minimally stressful for horses and could produce positive outcomes or emotions in horses [14,18,19]. Nevertheless, a recent study suggested that EAT does not increase equine well-being [20]. The conclusions of that study relied on HRV and the plasmatic concentrations of cortisol and oxytocin before, during, and after EAT sessions. The assessment of oxytocin concentrations in equine plasma through enzyme immunoassay requires an extraction phase, as described by Bienboire-Frosini et al. [38]. This procedure was not performed in the previously cited study [20]. However, the results of HRV in that study were in line with the results obtained in the present study. The results of the present study concerning both behavior and HRV suggested that horses experienced neither negative nor positive emotions during EAT activities. In addition, horses rapidly balanced their ANS after these sessions. Hence, we suggest that the horses involved in EAT perceive EAT activities as neutral rather than negative or positive.

The positive or negative indicators of emotions could be considered one limitation in this study. Snort has often been described as an indicator of negative emotions [32,33,34]; however, recently, some authors have described this behavior as a positive indicator [31]. HLM was also described as an indicator of negative emotions [14], although this behavior could also be related to the natural curiosity of horses. These findings highlight the need for further research on the use of behavioral indicators to study equine emotions, especially positive emotions. Nevertheless, in the present study, HRV was also assessed to complement the behavioral assessment. This tool has been commonly used to study stress or negative emotions, but positive emotions could also be studied through this analysis, because positive emotions create modifications in the activity of the sympathetic and parasympathetic systems [16,29]. 

Another limitation to this study was the variability of the activities performed in each phase. Activities such as brushing and cleaning the feet, as well as riding the horse or performing in-hand work, may have induced different reactions. The variability in the horses’ reactions to the different situations within each phase was not investigated in the present study, as the aim was to assess the overall reaction of horses to the preparation and working phases, and to assess whether EATs were perceived as positive or negative by the horses. Additionally, each group of patients in this study included patients with different illnesses, who may interact differently with the horses, producing negative or positive outcomes.

Previous studies on EAT have focused on the negative outcomes that EAT activities could represent for horses [14,19,20]. The results of these investigations suggested that there are no negative outcomes related to EAT activities for the studied animals. In addition, our results suggested that there were neither negative nor positive outcomes related to EAT for horses. Moreover, as described above, there were significant differences between patients in the PSY and the PSY+PHY groups, but these differences should be interpreted carefully, as the observed variables were within normal ranges [21]. These findings are of great importance, because they show that even if an event is not perceived as negative or does not induce a negative outcome for horses, a positive association with the event or a positive outcome should not be expected.

## 5. Conclusions

In conclusion, EAT was neither a negative nor a positive event for the horses in this study, as demonstrated by the horses’ behavior and HRV parameters. Behavioral and HRV changes were higher in horses involved in EATs with patients who had both physical and psychological therapy expectations than in horses involved in EATs with patients who had only psychological therapy expectations.

HRV is useful for measuring ANS activity, and thus assessing equine emotional balance during EAT.

Further research focusing on the positive aspects of these therapies for horses is required. This future research should focus on improving equine welfare in EAT, e.g., providing horses with positive stimulation and reinforcement, thus producing positive outcomes for the horses involved in EAT.

## Figures and Tables

**Table 1:** Disorders diagnosed in the included patients.

PSY	PHY + PSY
Autism spectrum disorder	Head injury with locomotor disability
Attachment disorder	Development deficit with locomotor disability
Schizophrenia	Pediatric spastic triplegia
Intellectual disability	Pitt–Hopkins syndrome
Anxiety	Post-traumatic physical rehabilitation
Attention deficit disorder	Trisomy 21 (Down syndrome)
Hyperactivity syndrome	
Depression	
Phobias with hallucination	

psychological (PSY) or both physical and psychological (PHY + PSY) expectations.

**Table 2:** Ethogram used for behavioral observation [14,31].

Behavior	Definition	Emotions
Ears pinned back (EPB)	Backward placement of the ears	Stress, irritation, or frustration; negative emotions
Head lateral movement (HLM)	Turning the head to one side or the other when no command is provided	Stress, irritation, or frustration; negative emotions
Snort	Short, powerful exhalation from the nostrils	Relaxation; positive emotions
Defecation	Elimination of excrement	Stress, irritation, or frustration; negative emotions

**Table 3:** Adapted description of heart rate variability (HRV) variables from Stucke et al., 2015 and Usui and Nishida, 2017 [23,28].

Variable	Definition	Interpretation
HR (beats per minute)	Heart rate mean	Overall variability of cardiac activity
VLF (%)	Very low frequency	Slow recovery component of the HRV (vagal tone)
LF/HF ratio	Low frequency/high frequency ratio	Representation of the balance between the sympathetic and parasympathetic nervous systems

**Table 4:** EAT effect on HRV: effect of phase.

Parameter	Phase	Mean ± SE	Median	Minimum	Maximum
HR (bpm)	Preparation	44.22 ± 0.95 ***	42.00	34.00	67.00
	Working	55.98 ± 1.62 ***	54.00	37.00	95.00
VLF (%)	Preparation	0.70 ± 0.19 *	0.30	0.03	7.44
	Working	0.74 ± 0.09 *	0.57	0.01	3.13
LF/HF Ratio	Preparation	2.71 ± 0.41 **	1.92	0.08	16.12
	Working	4.84 ± 0.57 **	3.39	0.22	15.13
ΔHR	Preparation	−0.46 ± 1.08	−3.00 ***	−11.00	31.00
	Working	11.40 ± 1.61	9.00 ***	−6.00	56.00
ΔVLF	Preparation	0.16 ± 0.21	−0.02 *	−1.08	7.19
	Working	0.21 ± 0.10	0.23 *	−0.98	2.21
ΔLF/HF Ratio	Preparation	−0.01 ± 0.46	−0.58 ***	−5.42	14.60
	Working	2.35 ± 0.58	1.52 ***	−6.64	17.07

* *p* < 0.05; ** *p* < 0.01; *** *p* < 0.001.
